# Supplementary material for: Mycousfurans A and B, Antibacterial Usnic Acid Congeners from the Fungus Mycosphaerella sp., Isolated from a Marine Sediment
Source: Mar Drugs. 2019 Jul 19;17(7):422. doi: 10.3390/md17070422 (PMC6669435; doi:10.3390/md17070422)

Supporting Information for

**Mycousfurans A-B, Antibacterial Usnic Acid Congeners from**

***Mycosphaerella* sp. Isolated from Marine Sediment**

Jihye Lee^1,2,3^, Jusung Lee^3^, Weihong Wang^3^, Inho Yang^4^, Sang-Jip Nam^2,^* and Heonjoong Kang^3,5,^*

^1^ Laboratories of Marine New Drugs, REDONE Seoul, Seoul 08594, Korea; jl3414@gmail.com (J.L.)

^2^ Department of Chemistry and Nanoscience, Ewha Womans University, Seoul 03760, Korea; jl3414@gmail.com (J.L.); [sjnam@ewha.ac.kr(S.-J.N.)](mailto:sjnam@ewha.ac.kr(S.-J.N.))

^3^ Laboratory of Marine Drugs, School of Earth and Environmental Sciences, Seoul National University, NS-80, Seoul 08826, Korea; jl3414@gmail.com (J.L.); [leejusung@snu.ac.kr](mailto:leejusung@snu.ac.kr) (J.L.); [pharmacy_china@hotmail.com](mailto:pharmacy_china@hotmail.com) (W.W.), [hjkang@snu.ac.kr](mailto:hjkang@snu.ac.kr) (H.K.)

^4^ Department of Convergence Study on the Ocean Science and Technology, Korea Maritime and Ocean University, Busan 49112, Korea; [ihyang@kmou.ac.kr](mailto:ihyang@kmou.ac.kr) (I. Y.)

^5^ Research Institute of Oceanography, Seoul National University, NS-80, Seoul 08826, Korea; hjkang@snu.ac.kr (H.K.)

***** Correspondence: sjnam@ewha.ac.kr (S.-J.N.); Tel.: +82-2-3277-6805 (S.-J.N.), hjkang@snu.ac.kr (H.K.); Tel.: +82-2-880-5730 (H.K.)

**Table of Contents**

Figure S1 ^1^H NMR spectrum (700 MHz, CDCl_3_) of mycousfuran A (**1**) ………………………………………………………………· **S3**

Figure S2 ^13^C NMR spectrum (175 MHz, CDCl_3_) of mycousfuran A (**1**) ……………………………………………………………… **S4**

Figure S3 HSQC spectrum (700 MHz, CDCl_3_) of mycousfuran A (**1**) ………………………………………………………………···· **S5**

Figure S4 HMBC spectrum (700 MHz, CDCl_3_) of mycousfuran A (**1**) …………………………………………………···…………… **S6**

Figure S5 NOESY spectrum (700 MHz, CDCl_3_) of mycousfuran A (**1**) ……………………………………………………………… **S7**

Figure S6 ^1^H NMR spectrum (700 MHz, CDCl_3_) of mycousfuran B (**2**) ……………………………………………………………… **S8**

Figure S7 ^13^C NMR spectrum (175 MHz, CDCl_3_) of mycousfuran B (**2**) …………………………………………………………··· **S9**

Figure S8 HSQC spectrum (700 MHz, CDCl_3_) of mycousfuran B (**2**) ………………………………………………………………·· **S10**

Figure S9 HMBC spectrum (700 MHz, CDCl_3_) of mycousfuran B (**2**) ………………………………………………………………·· **S11**

**Figure S1** ^1^H NMR spectrum (700 MHz, CDCl_3_) of mycousfuran A (1)


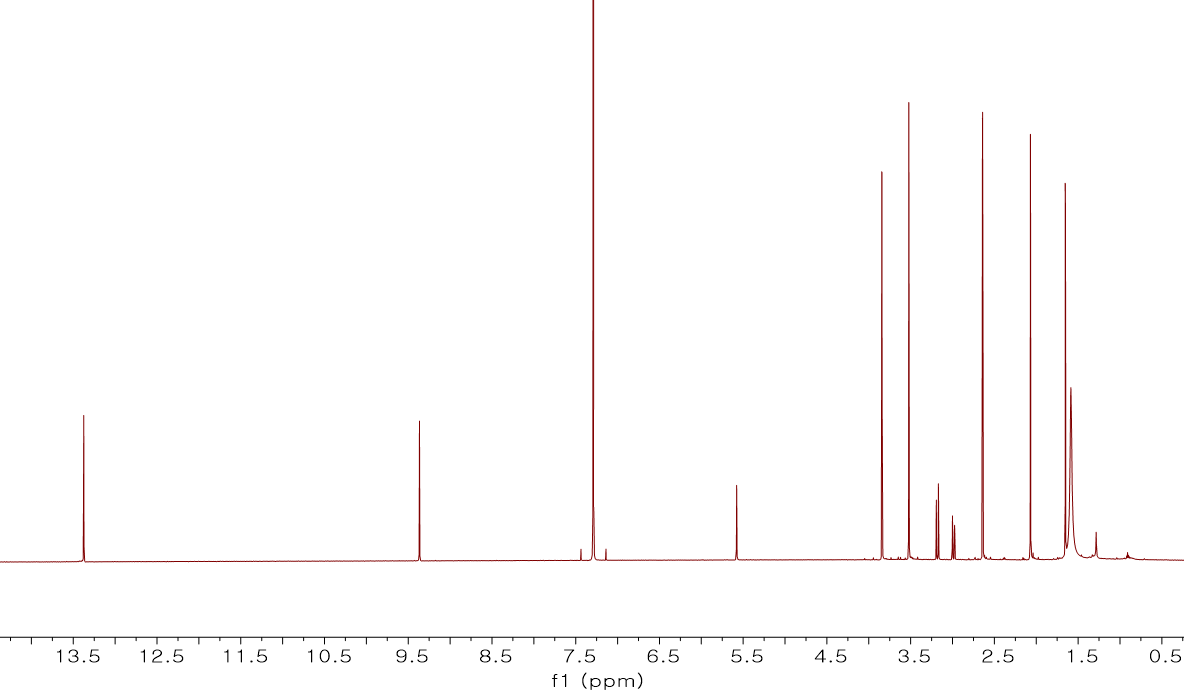


**Figure S2** ^13^C NMR spectrum (175 MHz, CDCl_3_) of mycousfuran A (**1**)


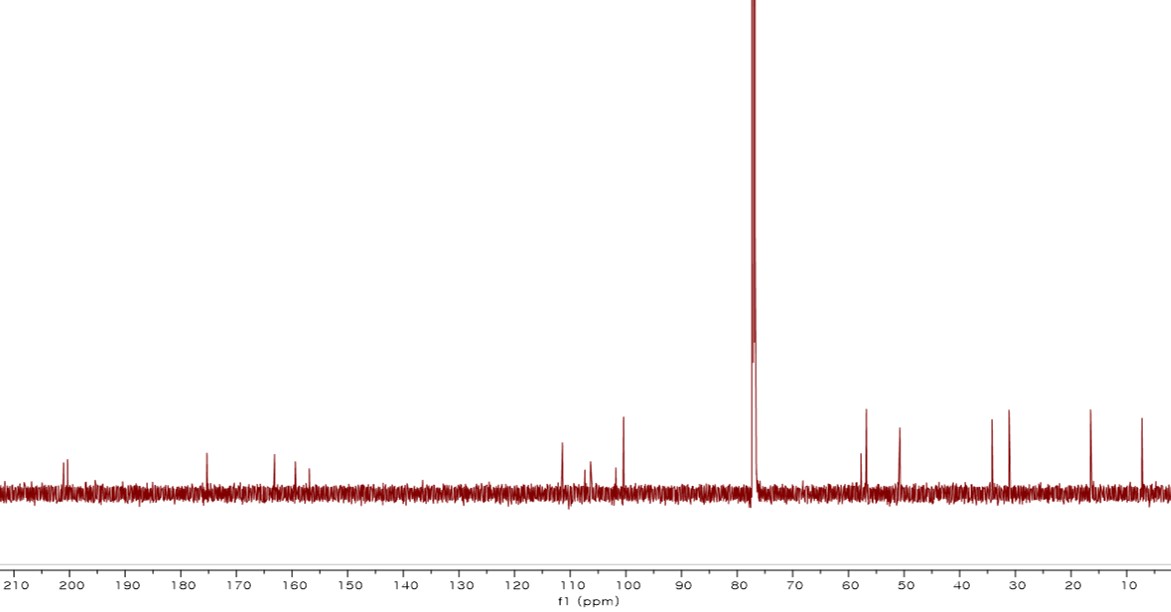


**Figure S3** HSQC spectrum (700 MHz, CDCl_3_) of mycousfuran A (**1**)


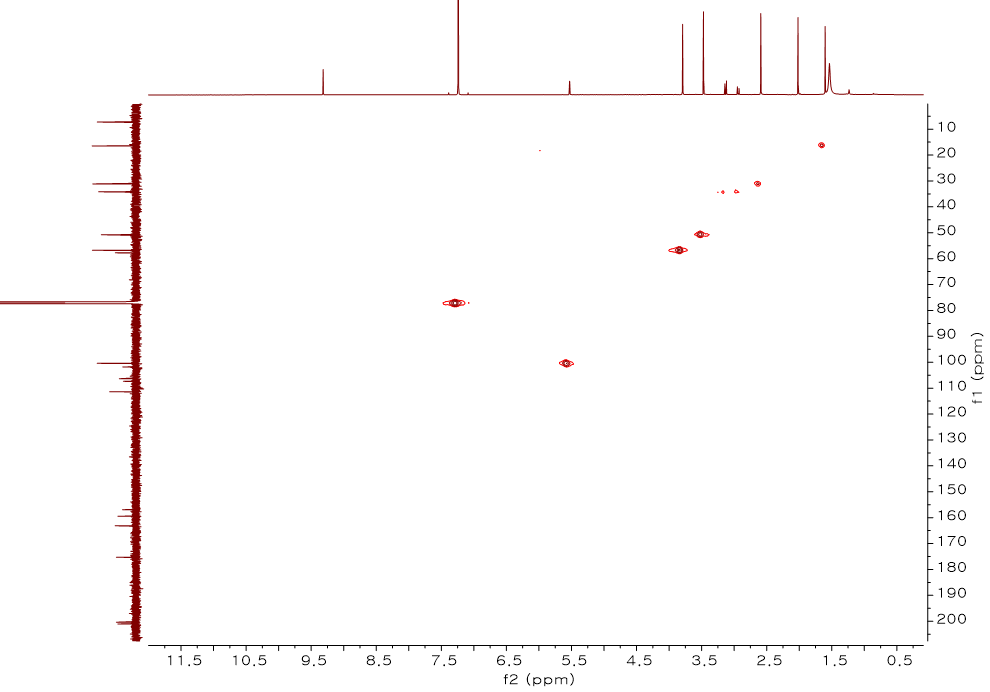


**Figure S4** HMBC spectrum (700 MHz, CDCl_3_) of mycousfuran A (**1**)

**
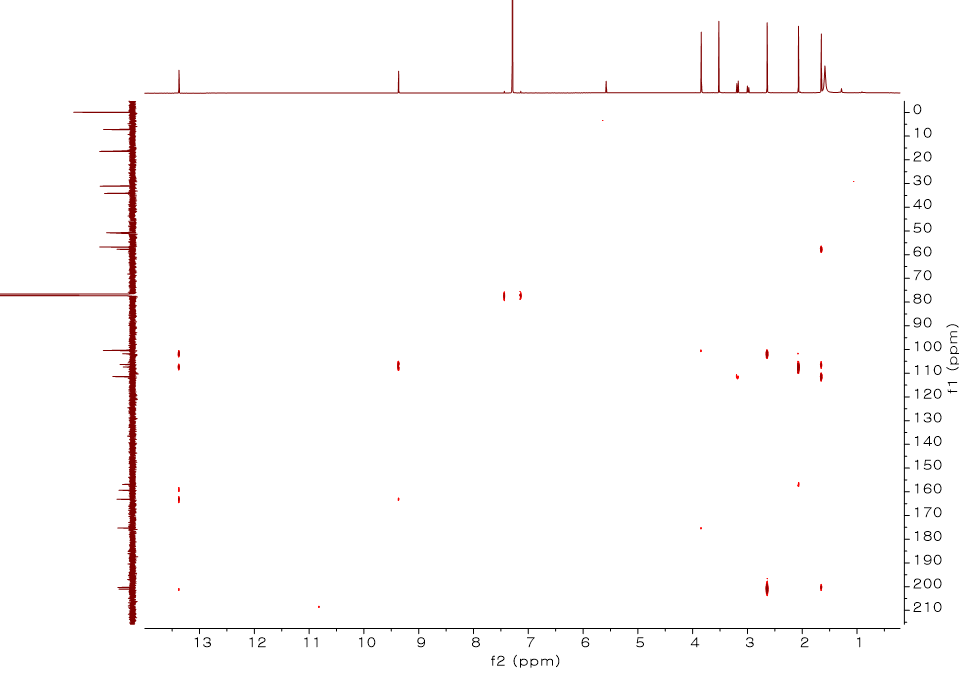
**

**Figure S5** NOESY spectrum (700 MHz, CDCl_3_) of mycousfuran A (**1**)

**
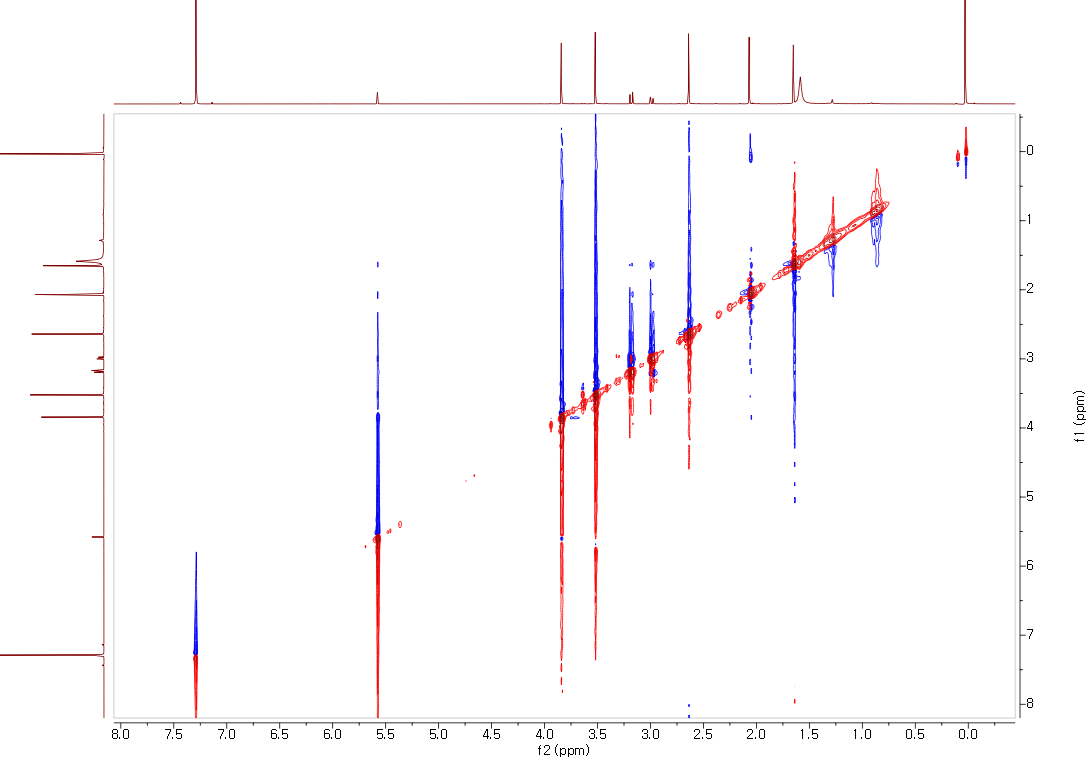
**

**Figure S6** ^1^H NMR spectrum (700 MHz, CDCl_3_) of mycousfuran B (**2**)


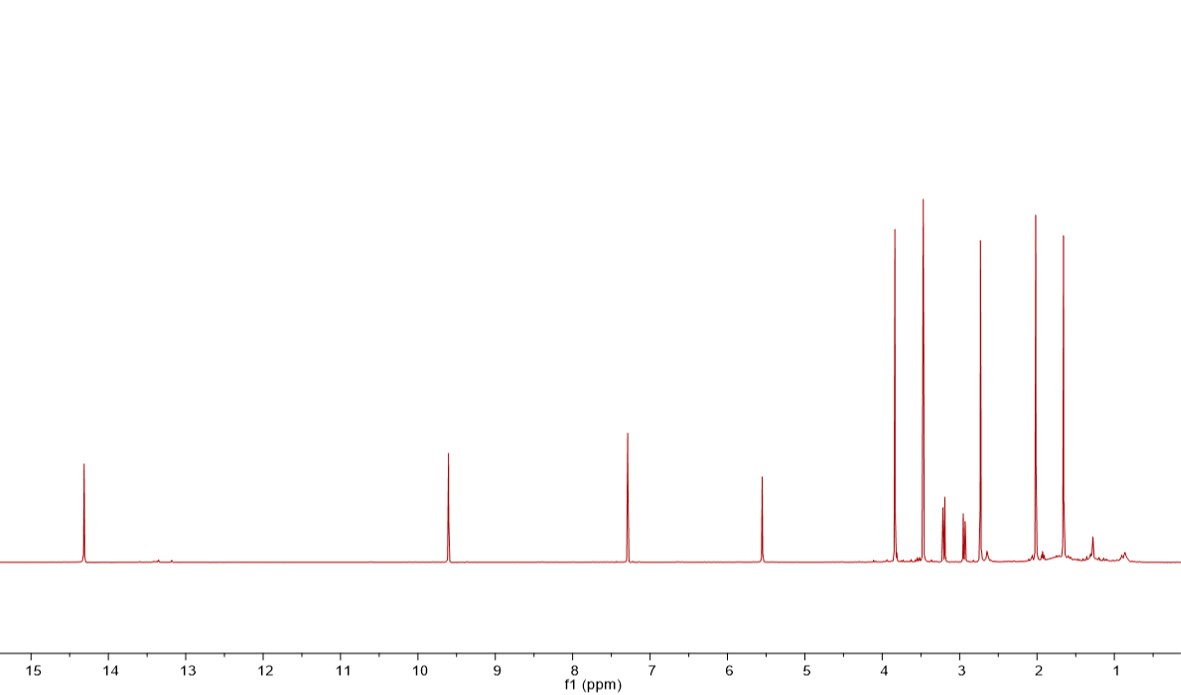


**Figure S7** ^13^C NMR spectrum (175 MHz, CDCl_3_) of mycousfuran B (**2**)

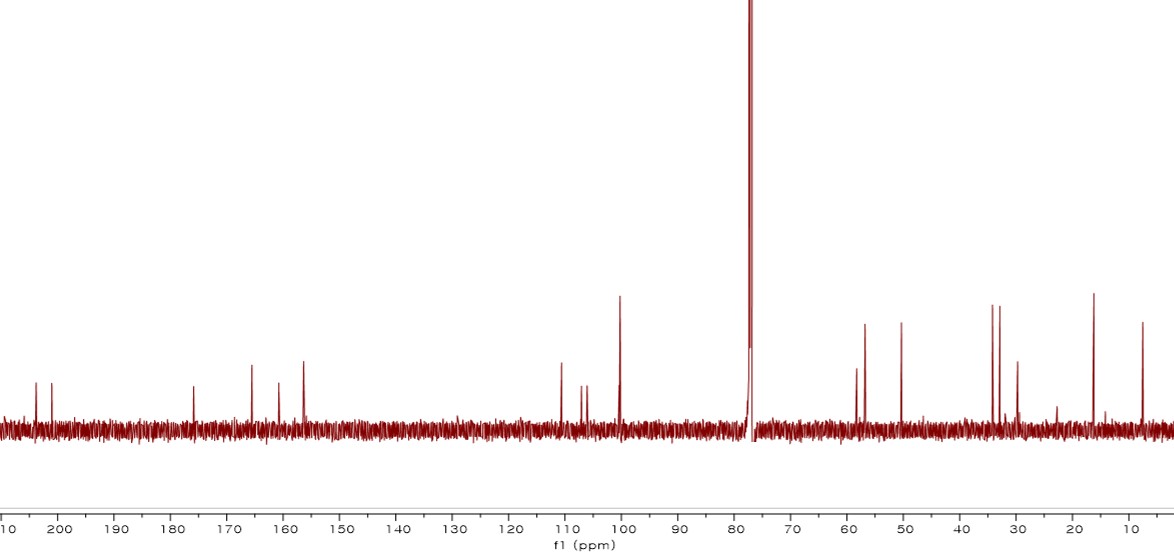


**Figure S8** HSQC spectrum (700 MHz, CDCl_3_) of mycousfuran B (**2**)


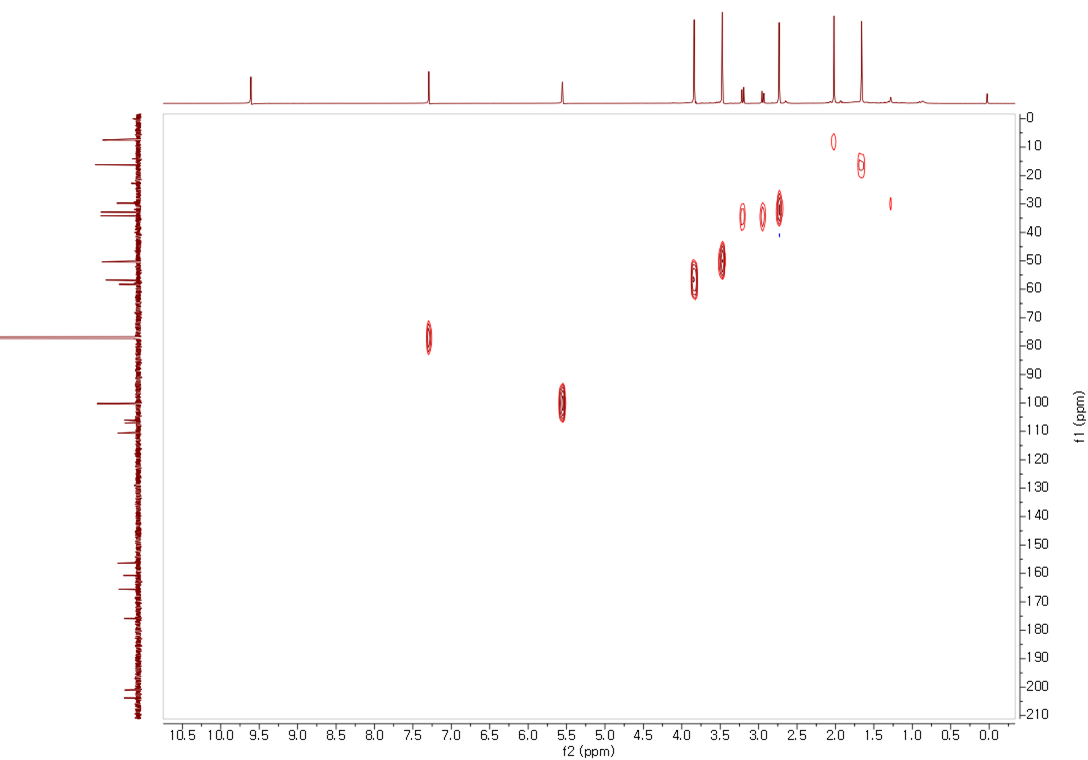


**Figure S9** HMBC spectrum (700 MHz, CDCl_3_) of mycousfuran B (**2**)


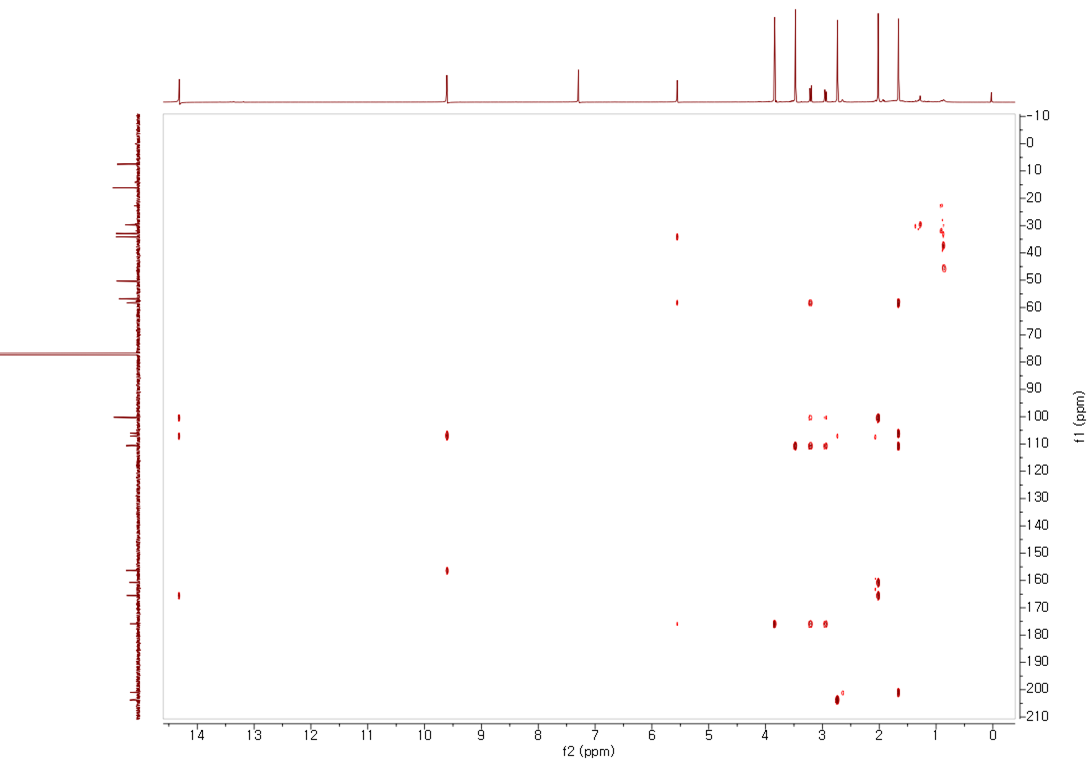

Supplement: Supplementary file 1 [file marinedrugs-17-00422-s001.zip › F8015_SI_Marine Drugs_sub.docx]
